# Nested PCR for detection of HSV-1 in oral mucosa

**DOI:** 10.4317/medoral.20630

**Published:** 2015-10-09

**Authors:** Miranda-Masoumeh Jalouli, Jamshid Jalouli, Bengt Hasséus, Jenny Öhman, Jan-Michaél Hirsch, Lars Sand

**Affiliations:** 1 Department of Surgical Sciences, Oral and Maxillofacial Surgery, Uppsala University, Sweden; 2 Department of Oral Medicine and Pathology, Institute of Odontology, Sahlgrenska Academy at the University of Gothenburg, Gothenburg, Sweden

## Abstract

**Background:**

It has been estimated that 15%-20% of human tumours are driven by infection and inflammation, and viral infections play an important role in malignant transformation. The evidence that herpes simplex virus type 1 (HSV-1) could be involved in the aetiology of oral cancer varies from weak to persuasive. This study aimed to investigate by nested PCR (NPCR) the prevalence of HSV-1 in samples from normal oral mucosa, oral leukoplakia, and oral squamous cell carcinoma (OSCC).

**Material and Methods:**

We investigated the prevalence of HSV-1 in biopsies obtained from 26 fresh, normal oral mucosa from healthy volunteers as well as 53 oral leukoplakia and 27 OSCC paraffin-embedded samples. DNA was extracted from the specimens and investigated for the presence of HSV-1 by nested polymerase chain reaction (NPCR) and DNA sequencing.

**Results:**

HSV-1 was detected in 14 (54%) of the healthy samples, in 19 (36%) of the oral leukoplakia samples, and in 14 (52%) of the OSCC samples. The differences were not statistically significant.

**Conclusions:**

We observed a high incidence of HSV-1 in healthy oral mucosa, oral leukoplakia, and OSCC tissues. Thus, no connection between OSCC development and presence of HSV-1 was detected.

**Key words:**HSV-1, nested PCR, PCR.

## Introduction

The World Health Organization (WHO) predicts a continuing worldwide increase in the incidence of oral cancer, extending this trend into the next several decades (1). It is generally accepted that oral carcinogenesis is a multistep process, and the main etiological factors in Western countries include alcohol consumption, tobacco use, and poor oral hygiene, which act on genetically susceptible individuals (2). Besides established risk factors for the development of oral cancer and pre-cancer, there are a number of factors that are contributory, such as viral infections (3). It has been estimated that 15%–20% of human tumours are driven by infection and inflammation (4).

HSV-1 is among the most common infectious agents in humans (5). While the infections are frequently asymptomatic, they can produce a variety of signs and symptoms, and these include oral or perioral lesions (6). HSV is a cytotoxic virus that readily infects and kills human cells, including cancer cells (7). Some previous studies have shown that the prevalence of infection with HSV-1 varies widely in esophageal carcinoma, depending on, for example, the population, type of specimen, racial difference, and sensitivity of the detection technique or the study methods (8).

In a review study from 1990 to 2009, herpes viruses (HSV-1) were associated with carcinogenesis, and antibody levels to HSV-1 and HSV-2 were higher in oral cancer patients compared to controls (4). In a study on rats by Hirsch *et al*. (9), it was demonstrated that the incidence of malignant tumours was significantly higher in rats exposed to snuff and HSV-1+ snuff than in control animals. Another study showed an association between the herpes virus and head and neck carcinomas (10). Further, HSV-1 and HSV-2 were shown to cause amplification of an HPV cell line, indicating the possibility of HSV-1 involvement in the integration in and amplification of HPV in the host cell (11).

Identification of the virus is dependent on specific techniques. There are currently four such methods, listed in order of increasing sensitivity: identification of the virus by electron microscopy; staining of the capsule antigen with immunohistochemistry techniques; identification of viral DNA by in situ hybridization; and most sensitive of all, the polymerase chain reaction (PCR) technique, based on DNA amplification (12).

In this study we aimed to investigate the presence of HSV-1 DNA by nested PCR (NPCR) in clinically healthy oral mucosa, oral leukoplakia, and oral squamous cell carcinoma (OSCC).

## Material and Methods

- Tissue specimens.

The study was carried out on 53 cases of oral leukoplakia and 27 cases of OSCC. The formal infixed, paraffin-embedded tissue sections were obtained from the Department of Oral Pathology, GothenburgUniversity. The patients’ age ranged from 23 to 93 years (34 male, 19 female, mean=65 years, SD=15) in patients with oral leukoplakia, and from 20 to 84 years (20 male, 7 female, mean=63 years, SD=16) in patients with OSCC.

Twenty-six clinically healthy volunteers, 14 male and 12 female, with mean ages 62±SD 15 years ( Table 1) were also included. Local anaesthesia (lidocaine 20 mg/mL+12.5 μg adrenaline; AstraZeneca, Södertälje, Sweden) was used to obtain biopsy specimens, which were taken from normal oral mucosa of clinically healthy Swedish volunteers, during dentoalveolar surgery. Immediately afterwards, the biopsy specimens were rinsed twice in buffered saline. The specimens were placed in 99% alcohol and kept at room temperature for 24 hours before being stored at –20°C until analysed. The specimens were obtained from the Department of Oral and Maxillofacial Surgery at Uppsala University.

**Table 1:**
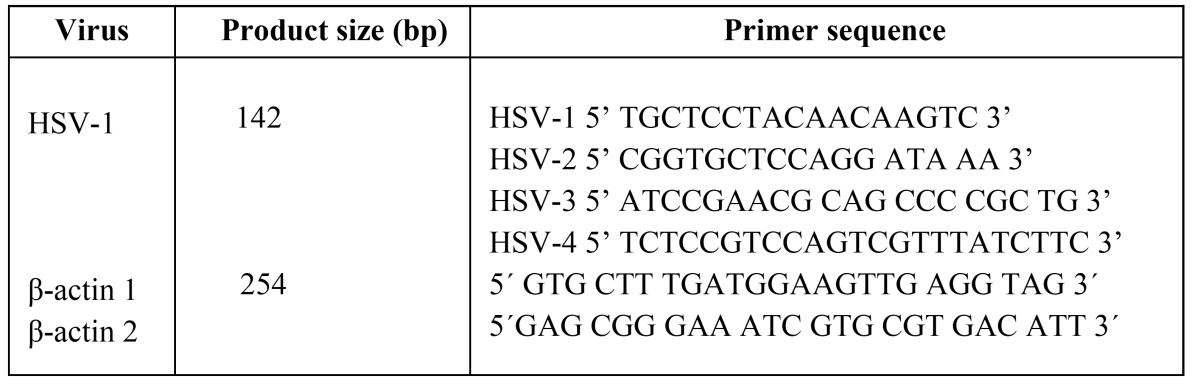
The primer sequences used in the PCR reactions.

Informed consent was obtained from all volunteers. The volunteers had no ongoing history of diseases associated with HSV-1. The study was approved by the Ethics Committee of Uppsala University. DNA extraction

- Paraffin-embedded tissue.

All of the tissue specimens were fixed with formalin and embedded in paraffin. Ten 5 μm sections were cut from each paraffin block. Paraffin was dissolved with xylene, and digestion of tissues was done with proteinase K. DNA was purified by sequential phenol/chloroform extraction and salt/ethanol precipitation. DNA was dissolved in TE buffer (10 mM Tris, 1 mM EDTA, pH 8.0). DNA concentrations and DNA quality were measured using Nano drop. All DNA samples were tested by PCR with a housekeeping gene and were positive with β-actin.

- Fresh tissue.

Total DNA was extracted from fresh oral biopsies using the QIAamp tissue DNA Mini Kit manufacturer’s protocol (QIAGEN, Hilden, Germany). Briefly, tissue samples were weighed, cut into small pieces, and incubated at 56°C by addition of 180 μL of ATL buffer supplied with 20 μL of proteinase K per 25 mg of sample. When tissues were completely lysed, a volume of 200 μL of lysate was transferred into a 2 mL micro centrifuge tube, and DNA extraction with QIAamp Mini spin columns was carried out using a QIAcube automate. Final elution of DNA extracted from tissue samples was performed with 200 μL of double-distilled water. DNA concentrations and DNA quality were measured using Nanodrop. All DNA samples were tested by PCR with a housekeeping gene and were positive with β-actin.

- HSV-1 DNA nested PCR assay

DNA extracted from fresh oral biopsies was used to amplify HSV-1 DNA by NPCR amplifications. All PCR reactions had positive and negative controls. The positive control for HSV-1 was monkey kidney cell lines, GMK (Gothenburg University, Gothenburg, Sweden). The primer sequences used in the PCR reactions are shown in  Table 2.

**Table 2:**
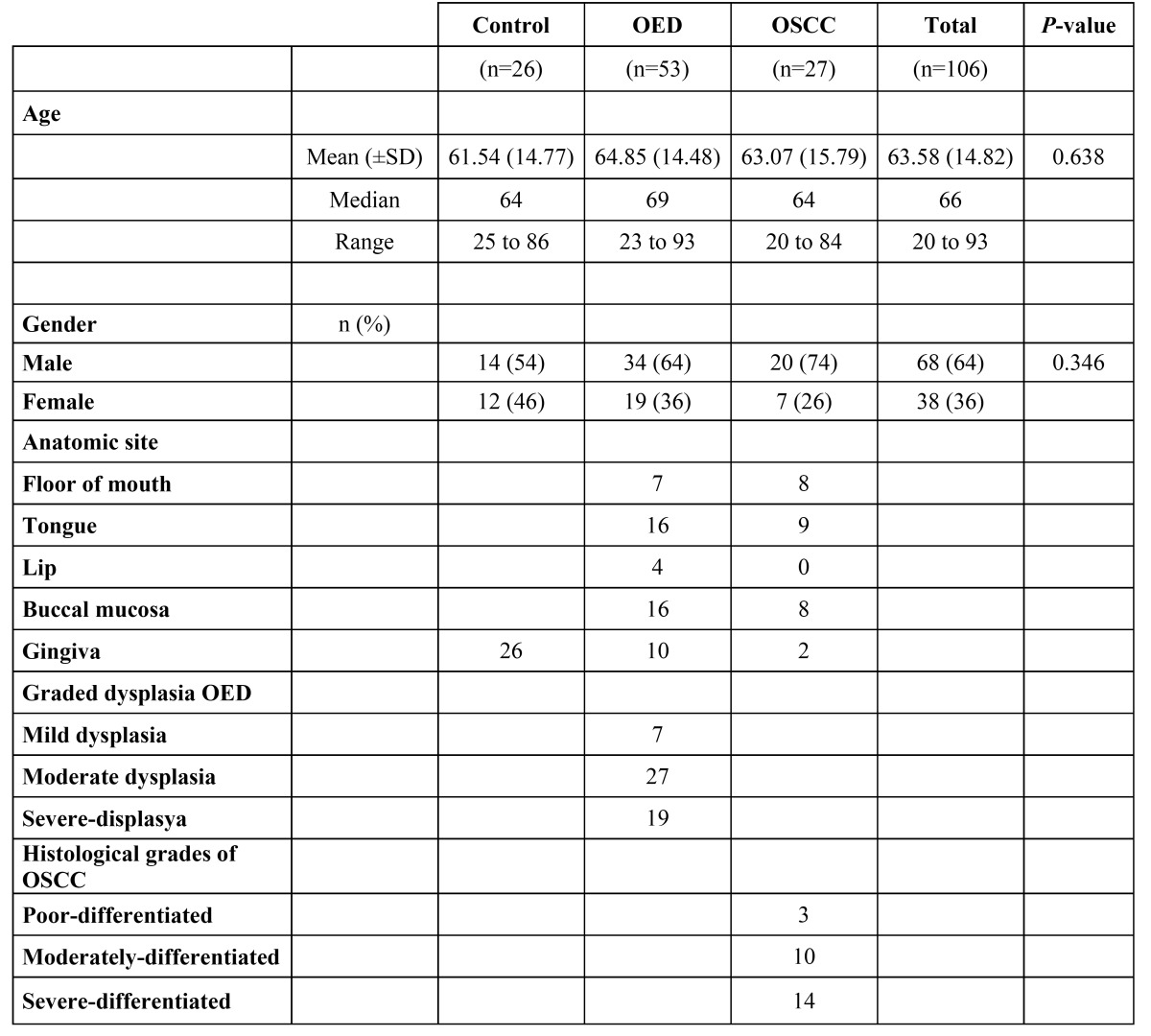
Clinical parameters of the leukoplakia, OSCC and healthy samples.

HSV-1 was amplified with primers HSV-1/HSV-2 in the first round and HSV-3/HSV-4 in the second round. The final products included a 142-bp fragment of HSV-1.

The PCR reaction mix contained 0.4 M of the appropriate primer (10 nmol, Applied Biosystems, Foster city, CA,USA), 1×PCR buffer (GeneAmp 10×PCR Buffer II, Applied Biosystems), 200 mM of each dNTP (GeneAmp, dNTP Mix with dTTP, Applied Biosystems), 1.25 units of Taq DNA polymerase (AmpliTaq Gold, 5 U/µl, Applied Biosystems), nuclease-free water, and 1.5 mM (HSV) and 2.5 mM Epstein–Barr virus (EBV) of MgCl2 (25 mM MgCl2 solution, Applied Biosystems), in a final volume of 25 µl. Viral DNA, human DNA, and reaction controls were included in each run. DNA amplification was performed in an automated thermal cycler (GeneAmp PCR system 2700, Applied Biosystems). Reactions were brought to 95ºC for 10 min, followed by thirty cycles consisting of a denaturing step for 30 s at 94ºC, an annealing step for 30 s at 50ºC (HSV-1 first round) or 60ºC (HSV-1 second round), and an extension step for 30 s at 72ºC. A final extension step at 72ºC was carried out for 5 min. A total of 2 µl of the first-round product was used in the second round of amplifications.

- Gel electrophoresis.

Aliquots of 15 µl of the PCR product were analysed on 2% agarose gel (DNA Agar, Marine Bio Products Inc., Quincy, MA, USA) containing 0.5 g/mol of ethidium bromide (Merck KGaA, Darmstadt, Germany) and visualized under ultraviolet light. The size of the amplified product was determined by comparison with a base-pair ladder size marker for HSV-1, (GeneRuler, 100bp, 50 bp DNA Ladder Plus, and Fermentas, St Leon-Rot, Germany).

- Sequencing of double-stranded DNA NPCR product .

PCR products from HSV-1 were sequenced with fluorescent dye-label led dideoxynucleotides and cycle sequencing methods utilizing the Big Dye Terminator Cycle Sequencing Kit (PE Applied Biosystems). Sequencing products were purified of unincorporated dye-label led dideoxynucleotides by processing through Centri-Sep spin columns (PE Applied Biosystems). Sequence analysis was automatically performed on the ABI PRISM 310 Genetic Analyzer (PE Applied Biosystems), and we used the Basic Local Alignment Search Tool (BLAST) to compare sequencing results from HSV-1 nested PCR products.

- Statistical analysis.

Statistical analyses were performed using the SPSS software package (SPSS for Windows, version 16.0; SPSS, Inc., Chicago, IL, USA). Age differences were investigated using Student’s t-test, and gender analysis was performed with Fisher exact test. McNemar’s test was used to compare paired proportions.

## Results

- Study groups

A total of 26 healthy, fresh oral mucosa samples, and 53 and 27 paraffin-embedded leukoplakia and OSCC samples, respectively, were analysed for detection of HSV-1 by PCR and NPCR methods. The cases and healthy samples were statistically compared with respect to gender and mean age. There was no significant difference between male and females with respect to staging and grading among the groups ( Table 2). Anatomic sites in leukoplakia, OSCC and healthy specimens are shown in  Table 2. Grades of dysplasia in leukoplakia and histological grades of OSCC are also shown in  Table 2.

- Prevalence of HSV-1.

Fourteen (54%) of the healthy samples, 19 (36%) of leukoplakias, and 14 (52%) of OSCC samples were HSV-1 positive. We found a higher prevalence of HSV-1 in normal and OSCC tissues compared to the leukoplakia samples, although it was not statistically significant (*p*=0.232) (Table 3).

HSV-1 was detected in 11 (41%) of the OSCC samples from males compared to 3 (11%) OSCC samples from females. This difference was statistically significant (*p*<0.05) ( Table 3).

**Table 3:**
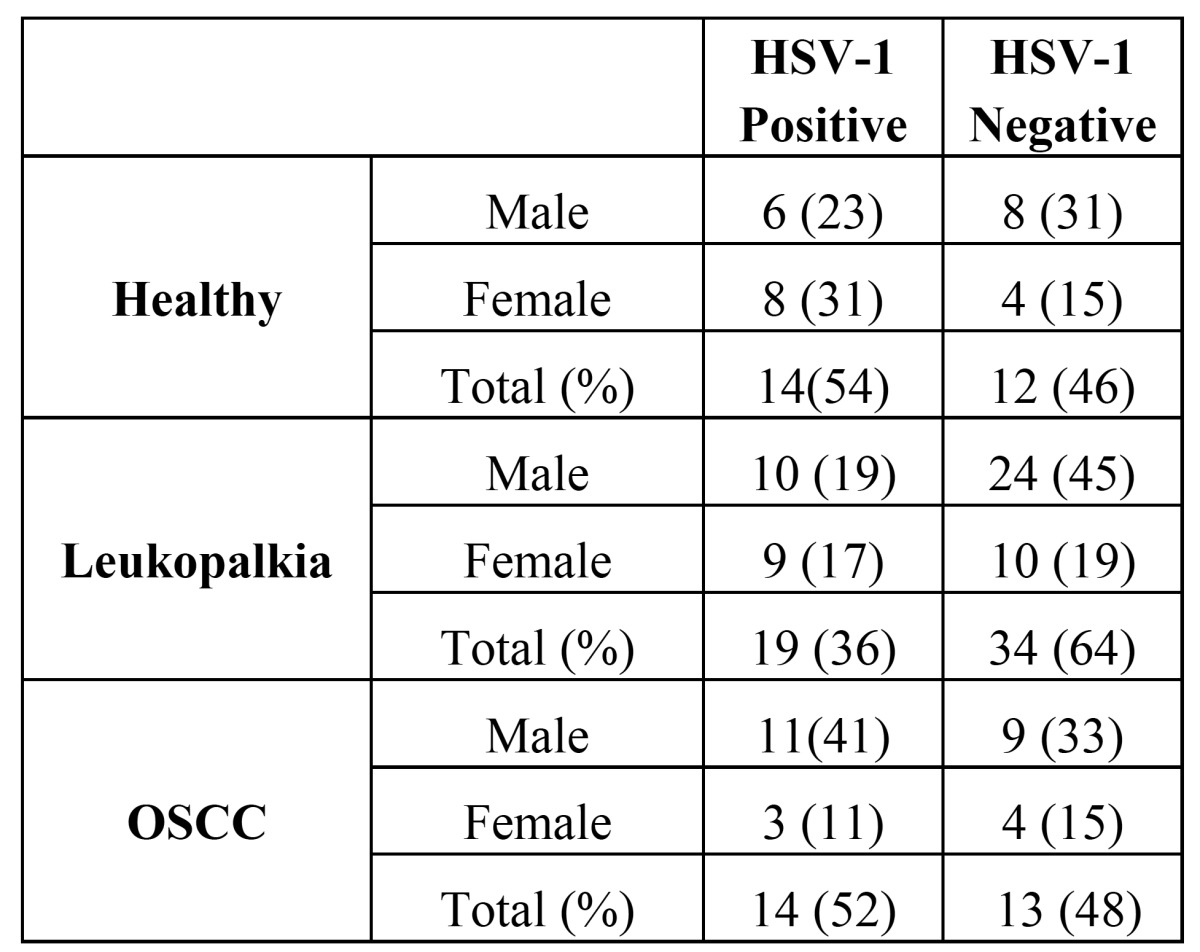
Prevalence of HSV-1 in leukoplakia, OSCC and healthy samples.

No statistically significant differences were found between the male and female patients with leukoplakia and healthy with regard to HSV-1 prevalence ( Table 3).

Presence of HSV-1 in different anatomic sites in leukoplakia, OSCC and healthy specimen are shown in  Table 4. Grades of dysplasia in leukoplakia and histological grades of OSCC are also shown in  Table 4.

**Table 4:**
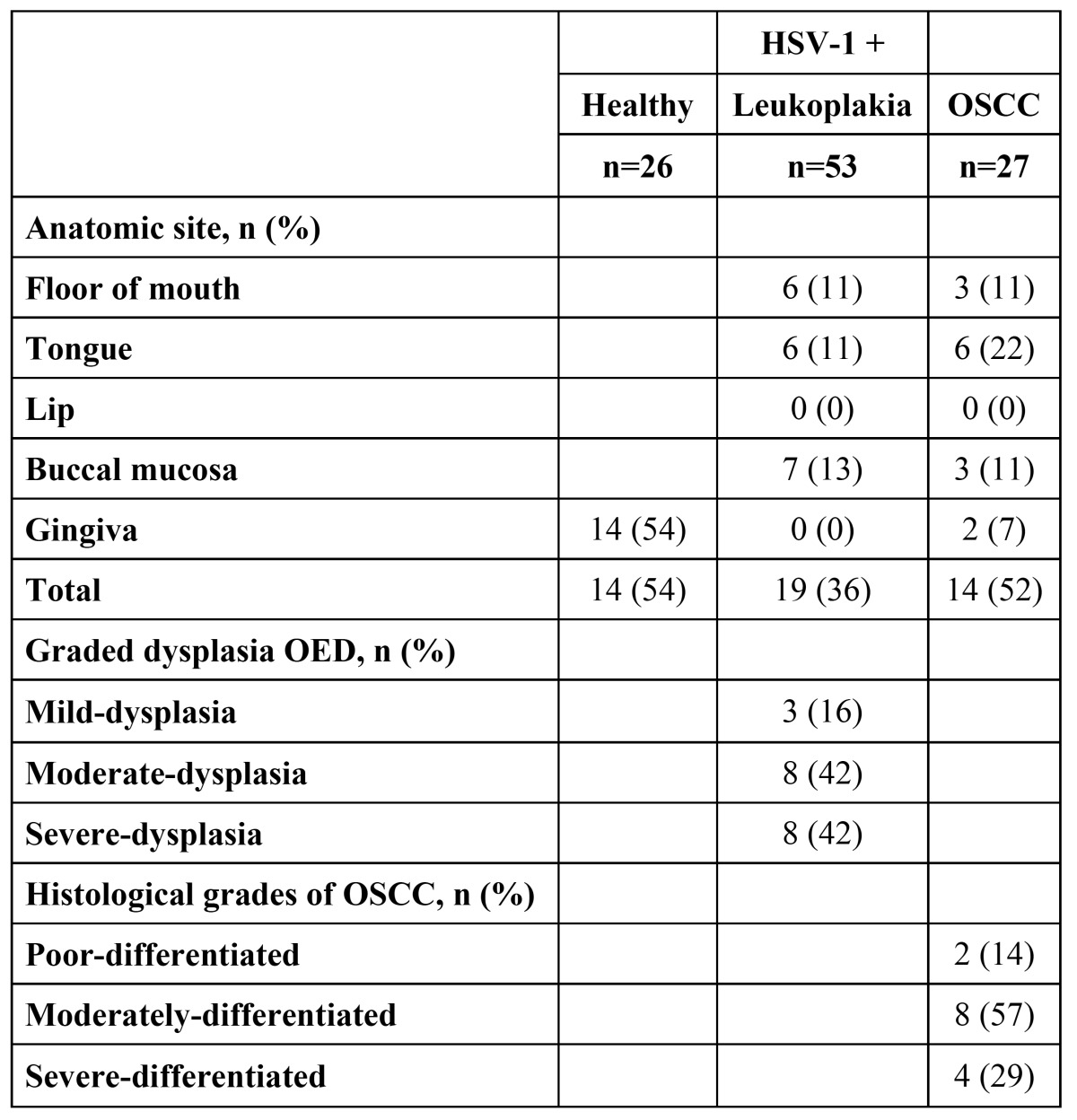
Presence of HSV-1 in different anatomic sites and histologic grading.

- Sequencing.

We used Basic Local Alignment Search Tool (BLAST) to compare sequencing result from HSV PCR product with nucleotide or protein sequences to sequence databases. The Basic Local Alignment Search Tool (BLAST) finds regions of local similarity between sequences.

## Discussion

Some viruses have the capacity to elude the immune system and remain latent. The mechanism to remain latent in the host depends on the type of virus, as EBV and HPV usually persist by either integrating into the host genome or by remaining episomal (13-15). The role of viral infection, the specific identification of tissues that harbour these viruses, and the way viruses persist in normal and tumour tissues are not fully elucidated (16). In the present study, normal oral mucosa, leukoplakia, and OSCC specimens were investigated by NPCR for the presence of HSV-1. We found higher prevalence of HSV-1 in the normal and OSCC samples compared with leukoplakia, although it was not statistically significant.

It has been shown that up to 90% of the general population have antibodies against HSV (17), and Miller *et al*. concluded that shedding of HSV-1 is present at many intraoral sites, for brief periods, at copy numbers sufficient to be transmitted, even in seronegative individuals (18).

In a review study from 2010, Meurman claimed that HSV infections link statistically with oral carcinogenesis and that antibody levels to HSV-1 and HSV-2 are increased in OSCC patients (19). In a population-based study from the USA, the authors concluded that HSV-1 may enhance the development of OSCC (20), and in another study HSV-1 antibody was associated with a slightly increased risk of head and neck cancer, although not statistically significantly (21).

However, another American study came to the opposite conclusion, where the risk of developing OSCC was not significantly increased in those with HSV-1 or HSV-2 antibodies compared to HSV-negative patients (22). In a study on OSCC by Jalouli *et al*., comparing eight different countries, the overall prevalence of HSV-1 was 15% with a huge span from 0% in the USA and India to 55% in the UK. There was a statistically significantly higher HSV-1 prevalence in the industrialized countries. However, no firm conclusions could be drawn regarding HSV-1, ethnicity, socioeconomic status, and OSCC development in that study (23,24). In two other studies conducted by our research group on specimens from India and Sudan, low HSV-1 prevalence was seen in OSCC (25,26).

There are several methods with varying sensitivity and specificity that can be used to detect HSV-1. Nested PCR method is a sensitive and useful tool compared with single PCR. A study suggests that the nested PCR is able to diagnose HSV infection (27). This method may detect even small copies of HSV-DNA, and our findings may result from the use of this high-sensitivity nested PCR assay.

In the present study, we found a high prevalence of HSV-1 in all study groups. However, whether HSV-1 plays an active role in OSCC development or is only a passive bystander in the decreased local immune-deficient tumour area remains unclear. Thus, it is still not clear what role HSV-1 plays in OSCC development.

In conclusion, we found that HSV-1 infection was not statistically associated with an increased risk of OSCC.
